# Editorial: Medicinal Plants in the Treatment of Myocardial Injury and Vascular Diseases

**DOI:** 10.3389/fphar.2022.879557

**Published:** 2022-04-01

**Authors:** Mas Rizky AA Syamsunarno, Zakiah Jubri, Yue Liu, Yusof Kamisah

**Affiliations:** ^1^ Department of Biomedical Sciences, Faculty of Medicine, Universitas Padjadjaran, Bandung, Indonesia; ^2^ Department of Biochemistry, Faculty of Medicine, Universiti Kebangsaan Malaysia, Kuala Lumpur, Malaysia; ^3^ Cardiovacular Health Research Group, Faculty of Medicine, Universiti Kebangsaan Malaysia, Kuala Lumpur, Malaysia; ^4^ Cardiovascular Diseases Center, Xiyuan Hospital, Beijing, China; ^5^ Department of Pharmacology, Faculty of Medicine Universiti Kebangaan Malaysia, Kuala Lumpur, Malaysia

**Keywords:** traditional medicine, ethnopharmacology, heart, vascular, traditional Chinese medicine

Cardiovascular diseases are the number one killer worldwide; approximately 17.9 million deaths were attributed to cardiovascular diseases in 2019, accounting for 32% of all global mortalities. The most common cardiovascular diseases are hypertension, atherosclerosis, heart failure, and ischemic heart disease (World Health Organization, 2021). These diseases are managed pharmacologically using modern medicines, such as statins (Bruikman et al., 2017), angiotensin-converting enzyme inhibitors, diuretics (Hui, 2020; Lin and Fang, 2020), and aspirin (Wan et al., 2020). However, the use of these drugs is not without several adverse effects. Because of this awareness, several studies have been conducted in search of alternative medicines, especially those from plants.

Approximately 24% of drugs approved by the United States Food and Drug Administration and other drug regulatory bodies from 1981 to September 2019 originated from natural sources, and approximately 12% of these newly approved drugs derived from natural products were cardiovascular drugs (Newman and Cragg, 2020). This development indicates that natural products, such as plants and microbes, are one of the key sources of new drugs. Many plants have potential medicinal values, and most of them have been used traditionally for various ailments since ancient times. Traditional medicines play a crucial role in the development of new drugs, many of which have progressed into orderly-regulated systems of medicine, such as traditional Chinese medicine (Yuan et al., 2016).

**FIGURE 1:**
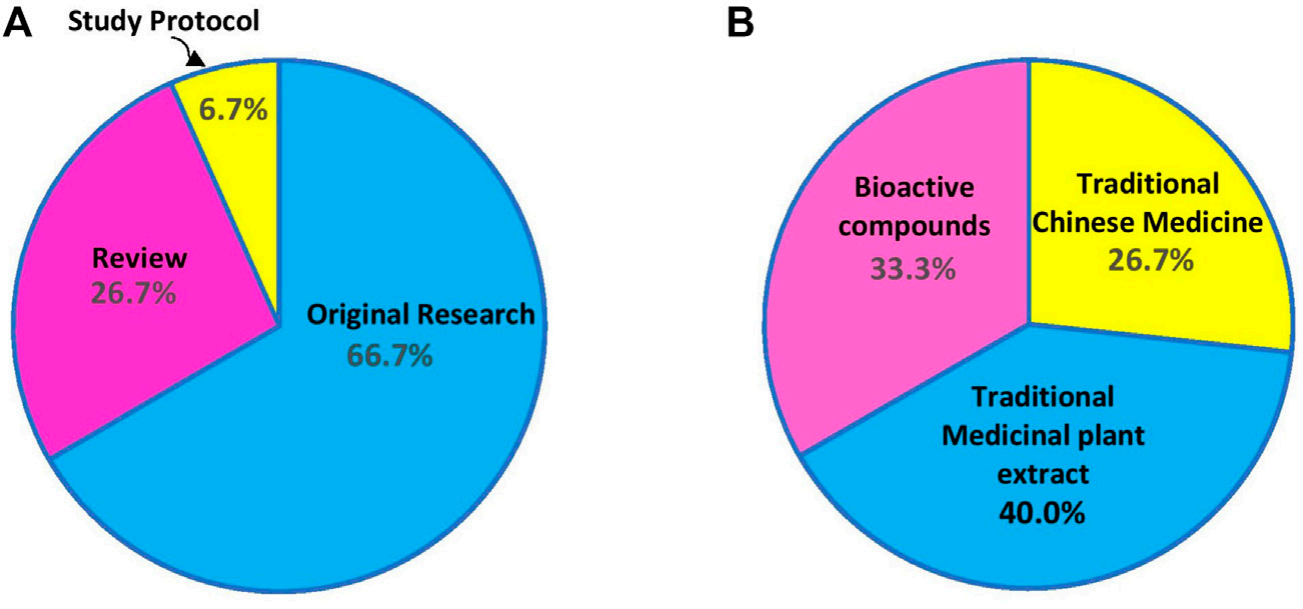
**(A)** The proportion of article types and **(B)** the type of studied medicinal plants published in the Research Topic.

The Research Topic Medicinal plants in the treatment of myocardial injury and vascular diseases collected studies carried out on crude extracts (typically used in a traditional context) and isolated bioactive compounds for the treatment of myocardial injury and vascular disease, focusing on the possible mechanisms of action. It is a collection of 15 articles that explore the effects of various medicinal plants against cardiovascular disease. More than two-thirds of the articles were original research articles and study protocol, and the remaining were reviews (Figure 1A), suggesting that research has actively explored medicinal plants in search of new drugs against cardiovascular diseases. The most common focus of articles submitted to the Research Topic was traditional medicinal plant extracts, followed by traditional Chinese medicines (Figure 1B). One-third of the articles studied bioactive compounds isolated from medicinal plants.

Danlou tablet is a commercially available traditional Chinese medicine composed of 10 herbs. Wang et al. and Liu et al. investigated its effects on atherosclerosis using apolipoprotein E-deficient (ApoE^−/−^) mice (an atherosclerotic mouse model) fed a high-fat diet. The medication improved the blood lipid profile, reduced blood inflammatory biomarkers, and decreased lipid deposition and fibrous plaque formation in atherosclerotic plaque. Its protective mechanism was most likely the activation of autophagy mediated by the phosphatidylinositol 3-kinase/protein kinase B/mammalian target of rapamycin (PI3K/Akt/mTOR) signaling pathway in vascular adventitial fibroblasts (Wang et al.) and macrophages (Liu et al.). Additionally, Liu et al. reported reduced CD68^+^ macrophage infiltration and inflammatory factor expression in the plaque.

Exposure to secondhand tobacco smoke that contains nicotine can lead to severe health consequences, such as an increased risk of vascular dysfunction (Whitehead et al., 2021). Md Salleh et al. reported that *Piper sarmentosum* Roxb. protected against nicotine-induced vascular endothelial dysfunction *in vitro*. They observed that the plant extract promoted vasorelaxation by enhancing vascular nitric oxide and antioxidant levels in nicotine-administered rats. Kim et al. investigated the effects of the seaweed *Codium fragile* (Suringer) Hariot on thrombosis and platelet aggregation by suppressing the activation of sarcoma tyrosine-protein kinase, spleen tyrosine kinase, phospholipase Cγ2, PI3K, Akt, and mitogen-activated protein kinases (MAPKs). The suppression inhibited the signaling of αIIbβ3 integrin, a primary mediator of platelet aggregation. Phytol detected in the seaweed may be the bioactive compound responsible for the observed effects.

In addition, oroxylin A purified from *Scutellaria baicalensis* Georgi conferred beneficial effects against ischemia after artery blockage due to peripheral artery disease (Zhang et al.). It attenuated tissue injury and promoted perfusion recovery, angiogenesis, and endothelial cell migration, most likely by stimulating the T-box20/prokineticin 2 and Ras homolog gene family member A (RhoA)/Rho-associated coiled-coil kinase 2 signaling pathways. On the basis of these findings, oroxylin A could be developed as a drug candidate for treating medical problems related to peripheral artery disease. Additionally, Vijakumaran et al. described the effects of hydroxytyrosol, a polyphenol from the olive plant (*Olea europaea* L.), on the progression of intimal hyperplasia in a scoping review. Hydroxytyrosol has an anti-inflammatory effect, which prevents intimal hyperplasia arising from endothelial inflammation. This effect is mediated by the upregulation of the PI3K/Akt/mTOR pathway; the inhibition of extracellular signal-regulated protein kinase pathway activation, which is involved in inflammation; and the inhibition of the stress-activated protein kinase pathway (also known as the c-Jun N-terminal kinase pathway), which is involved in apoptosis. Leong et al. described the anti-inflammatory effects of thymoquinone on atherosclerosis. Thymoquinone, a compound isolated from *Nigella sativa* L., modulates inflammatory events in atherosclerosis by suppressing the nuclear factor kappa-light-chain-enhancer of activated B cells and MAPK pathways.

Heart failure has become a serious global medical problem (Maraey et al., 2021). It is manifested as cardiac hypertrophy, the presence of fibrotic tissues, and impaired cardiac function (Siti et al., 2020). Various plants have demonstrated protective effects against heart failure in animal models. Huangqi Shengmai Yin, another traditional Chinese medicine, was studied in isoprenaline-induced heart failure in rats (Pan et al.). The medication reduced myocardial fibrosis and improved cardiac function in the rats, possibly by activating sirtuin 3 and inhibiting the transforming growth factor-β/Smad pathway. Moreover, *Parkia speciosa* Hassk., which is rich in flavonoids, protected against angiotensin II (Ang II)-induced cardiomyocyte hypertrophy *in vitro*, most likely by suppressing the activation of the MAPK signaling pathway, which is involved in inflammatory regulation and the Ang II/reactive oxygen species/nitric oxide axis (Siti et al.). Bunaim et al. also demonstrated that *Centella asiatica* (L.) Urb extract administered orally for 8 weeks prevented heart damage and the development of hypertension induced by N(G)-nitro-L-arginine methyl ester, a nitric oxide synthase inhibitor. The plant extract provided protection by inhibiting serum nitric oxide loss and myocardial angiotensin-converting enzyme activity, most likely through its antioxidant properties. Liu et al. described a study protocol for a basket trial involving BuqiTongluo granule for patients with ischemic stroke, stable angina pectoris, and diabetic peripheral neuropathy associated with qi deficiency and blood stasis syndrome. The treatment would be given for 6 weeks. This was the only clinical study included in the Research Topic.

The effects of two bioactive metabolites—ferruginol and aconitine—were studied in the hearts. Ferruginol, a terpenoid, is abundant in *Salvia spp.* plants, whereas aconitine, a diterpenoid, is found in *Aconitum spp.* Ferruginol afforded cardioprotection against doxorubin-induced cardiotoxicity by rescuing mitochondrial biogenesis and fatty acid oxidation via the sirtuin 1–peroxisome proliferator-activated receptor gamma coactivator-1α axis (Li et al.); this protection was observed as an improvement in cardiac function and a reduction in myocardial damage in rats. Qiu et al. also reported cardiotonic effects of repeated low-dose administration of aconitine in neonatal rat ventricular myocytes. These findings suggest that the metabolite promotes remodeling of mitochondrial function and thus increases energy metabolism, possibly via the AMP-activated protein kinase–optic atrophy 1–ATP synthase α-subunit pathway.

Syamsunarno et al. contributed an in-depth review of the effects of *Caesalpinia sappan* Linn. on the cardiovascular organs. They described the molecular mechanisms of the plant extract’s protective effects and its bioactive compounds—brazilin, sappanone A, and brazilein—against myocardial and vascular injuries. The protective effects of *Moringa oleifera* Lam. were also comprehensively reviewed, especially against cardiovascular-related metabolic syndrome (Alia et al.).

In conclusion, this Research Topic stipulates updated research studies and reviews that provide insights into the molecular mechanisms of the protective effects of various medicinal plants and their bioactive compounds against cardiovascular diseases. The bioactive compounds that have been reported in plants could potentially be developed as candidate drugs. Unfortunately, the Research Topic lacks findings from clinical studies. Thus, more studies in the clinical setting should be pursued to confirm the protective effects observed in the laboratory.
